# Acacetin improves cognitive function of APP/PS1 Alzheimer’s disease model mice via the NLRP3 inflammasome signaling pathway

**DOI:** 10.1515/tnsci-2022-0254

**Published:** 2022-10-31

**Authors:** Juan Bu, Yanmin Zhang, Yeledan Mahan, Shen Shi, Xuanxia Wu, Xiaoling Zhang, Zhaoxia Wang, Ling Zhou

**Affiliations:** Medical Research and Transformation Center, People’s Hospital of Xinjiang Uygur Autonomous Region, No. 91 Tianchi Rd., Urumqi, Xinjiang 830001, PR China; Scientific Research and Education Center, People’s Hospital of Xinjiang Uygur Autonomous Region, Urumqi, Xinjiang 830001, PR China; Disinfection and Infection Control Center, Center for Disease Control and Prevention of Xinjiang Uygur Autonomous Region, Urumqi, Xinjiang 830002, PR China

**Keywords:** acacetin, Alzheimer’s disease, NLRP3 inflammasome, spatial learning and memory, APP/PS1 mice

## Abstract

**Background:**

Acacetin (5,7-dihydroxy-4′-methoxyflavone), one of the main extractions from *Saussurea involucrata*, has anti-inflammatory effects. Our previous study found that acacetin inhibited the Nod-like receptor pyrin domain containing 3 (NLRP3) signaling pathway after cerebral ischemia–reperfusion injury. NLRP3 inflammasome plays a role in Alzheimer’s disease (AD) process. However, few studies have examined the effects of acacetin in AD.

**Methods:**

We randomly divided *APP swe/PS1dE9* double transgenic mice into acacetin group (intraperitoneal injection of 25 mg/kg acacetin) and AD model group (intraperitoneal injection of same volume of saline). C57BL/6 mice were selected as control group (same treatment with AD model group). After treating for 30 days, a Morris water maze test was conducted to evaluate spatial learning and memory of the mice. Senile plaque (SP) formation was evaluated by immunohistochemistry. NLRP3 inflammasome-related inflammatory factors and amyloid-β-42 were detected by Western blot or enzyme-linked immunosorbent assay.

**Results:**

Acacetin improved spatial learning and memory of AD mice and reduced APP/β expression, thereby decreasing SP formation in the brain. Acacetin also reduced the expression of NLRP3, cysteinyl aspartate-specific proteinase 1 (caspase-1), and interleukin-1β (IL-1β) and the release of inflammatory factors, tumor necrosis factor-α (TNF-α) and IL-1β.

**Conclusions:**

Acacetin improved the learning and memory abilities of AD mice and exerted a protective effect on AD by inhibiting the NLRP3 signaling pathway and reducing SP formation.

## 1 Introduction

Alzheimer’s disease (AD) is a neurodegenerative disorder that severely affects the quality of life in the elderly due to insidious onset, progressive intellectual disabilities, and personality changes [1,2]. According to a national cross-sectional study in 2020, there were 9.83 million people aged 60 and above with AD in China [3]. In addition, the annual treatment cost of AD in China was $167.74 billion in 2015, which is expected to be up to $1.89 trillion by 2050 [4]. The current medications approved for clinical use in China for AD are cholinesterase inhibitors – donepezil, rivastigmine, galantamine, the *N*-methyl-d-aspartate receptor-antagonist memantine, and Ginkgo biloba extract tablets [5]. However, there are no effective medications for halting AD disease progression. Therefore, it is of great significance to find safe and effective drugs for AD.

The etiology and pathogenesis of AD remain unclear. Currently, the main hypotheses about pathogenesis of AD are amyloid-cascade hypothesis, tau hypothesis, and neuroinflammation hypothesis [6–8]. The aggregation of amyloid-β (Aβ) in the brain is the most important pathological feature of AD. Amyloid precursor protein (APP) gene mutation or overexpression can cause the abnormal production and deposition of Aβ in the brain of the patients with AD. A large amount of Aβ accumulation was repeatedly detected in the brain tissue or cerebrospinal fluid of patients with AD. Later studies found that it is due to the neurotoxic peptide, which is directly related to the loss of spatial memory and the reduction of cognitive function produced by APP, which is over-cleaved by β-secretase and γ-secretase in turn [9]. Nucleotide-binding oligomerization domain-like receptors (NLRs) include Nod-like receptor pyrin domain containing 3 (NLRP3), Nod-like receptor pyrin domain containing 1 (NLRP1), NLR family CARD domain-containing protein 4 (NLRC4), and absent in melanoma 2 (AIM2). Among them, NLRP3 is the most characterized inflammasome. NLRP3 is a polyprotein compound consisting of NLRP3 scaffold, apoptosis-associated speck-like protein containing a CARD, and caspase-1 [10]. As an important component of innate immunity, NLRP3 plays a key role in immune response and disease [11].

In recent years, accumulated studies have shown that NLRP3 inflammasome plays an important role in the development and progression of AD. AD is characterized by the deposition of the Aβ in the brain. Aβ activates NLRP3 inflammasome by inducing the lysosomal damage [12]. Moreover, the inhibition of NLRP3 inflammasome can ameliorate AD progression, indicating that NLRP3 inflammasome may serve as a potential therapeutic target for AD [13].

Acacetin (5,7-dihydroxy-4′-methoxyflavone) is an *O*-methylated flavonoid with antioxidant and anti-inflammatory effects [14–16]. Acacetin has a protective effect on many diseases, but there are few studies related to AD. Acacetin extracted from the whole *Agastache rugosa* plant plays a protective role in AD for downregulating both APP and β-amyloid cleaving enzyme (BACE-1) protein expression, reducing Aβ production, and preventing the eye degeneration and behavioral abnormality in *Drosophila melanogaster* AD model by mediating the transcriptional regulation of APP and BACE-1 [17]. It is still unclear whether acacetin has a protective effect on AD. NLRP3 inflammasome is involved in AD process, we thus hypothesize that acacetin may protect against AD by inhibiting the expression of NLRP3 inflammasome.

Herein, APPswe/PS1dE9 (APP/PS1) double transgenic mice (with the poor spatial memory and poor coordination) were selected as the AD animal model and then treated with acacetin for 30 days. The spatial learning and memory were assessed by Morris Water Maze (MWM) test, senile plaques (SPs) were evaluated by immunohistochemistry, and the expressions of NLRP3 inflammasome signaling pathways were detected by Western blot (WB). Levels of Aβ-42, tumor necrosis factor-α (TNF-α), and interleukin-1β (IL-1β) were measured by enzyme-linked immunosorbent assay (ELISA). Hence, we aimed to elucidate whether acacetin has a protective effect for AD and whether the mechanism is related to the inhibition of NLRP3 inflammasome signaling pathways. Furthermore, our findings can provide evidence for acacetin as a potential drug for the treatment of AD.

## 2 Materials and methods

### 2.1 Animals

Twelve 3-month-old female APP/PS1 double transgenic mice and six 3-month-old female C57BL/6 mice were purchased from Henan Skbex Biotechnology Co., Ltd (China, License No.SCXK (Yu) 2020-0005). APP/PS1 double transgenic mice were randomly divided into two groups: the acacetin treatment group (given an intraperitoneal injection of 25 mg/kg acacetin every day for 30 days) and the AD model group (given an intraperitoneal injection of the same volume of 0.9% saline every day for 30 days). C57BL/6 mice were selected to be the control group (given the same treatment with the AD model group).


**Ethical approval:** The research related to animals’ use has been complied with all the relevant national regulations and institutional policies for the care and use of animals. All animal experiments were conducted according to the ethical guidelines of the Laboratory Animals Welfare and Ethics Committee of Xinjiang Uygur Autonomous Region Center for Disease Control and Prevention (Approval No. 2022007). All animal experiments were strictly in accordance with the Ministry of Science and Technology of the PRC Guide for the Care and Use of Laboratory Animals.

### 2.2 MWM

MWM test was conducted 1 week after drug treatment. The maze was carried out in a circular white pool 120 cm in diameter and 40 cm in depth. The pool was divided equally into four quadrants (designated NE, NW, SE, and SW) by using computer software. The pool was filled to a depth of 30 cm with water and made opaque with white non-toxic paint. The pool temperature was maintained at 20–21°C. The hidden platform was a 64 cm^2^ round, placed in the center of the SW quadrant, submerged 1 cm beneath the water surface, and remained in the same position throughout all trials and days.

#### 2.2.1 Spatial acquisition trial

The mice were transferred to the testing room and kept in an area where they could not see the pool and were left there to adjust to the new environment for at least 1 h before testing. The first day was the adaptation period (without the platform) and the mice were left in the water to adapt for 120 s. On the second to the sixth day (with the platform), the mice were placed into the water in different starting points of the pool wall. Track path length, escape latency, and time spent in each quadrant of each mouse. If the mice found the platform before 120 s had passed, they were removed from the pool immediately. If the mice found the platform after 120 s, then they were gently guided to the platform and allowed to stay for 20 s. Each mouse was tested four times a day.

#### 2.2.2 Probe trial

On the last day (seventh day), the platform was removed and the mice were let to start an exploration experiment for 120 s. The mice were put into the water from the middle point of the pool wall of the NE quadrant. The number of times and time required for the mice to cross the platform in the target quadrant were tracked.

### 2.3 Immunohistochemistry

Mice were perfused with 40 mL of normal saline and 4% paraformaldehyde through the left ventricle, respectively, and the brain was removed. The brain tissue was fixed overnight in 4% paraformaldehyde. After gradient dehydration, the brain tissue was embedded with OCT glue and cut into slices (hippocampus) of 10 μm thick in a Cryostat Microtome (Leica CM3050S, Leica Biosystems, Nussloch, Germany). The brain slices were immersed in a mixed solution of 0.01 M phosphate buffered saline (PBS) and glycerol (1:1) and frozen at −20°C. Then, 3% H_2_O_2_ was added to the brain slices to block endogenous peroxidase. After closing with goat serum, the brain slices were incubated with primary antibody APP/Aβ (1:100) (NAB228 Mouse mAb, Cell Signaling Technology, USA) overnight at 4°C. The brain slices were then washed three times with 0.01 M PBS and incubated with secondary antibody at room temperature for 1 h. The brain sections were stained with diaminobenzidine (DAB). Then, Aβ plaques were observed under a microscope (Olympus BX53, Olympus, Japan). IPP6.0 software was used to determine the optical density of APP/β.

### 2.4 WB assay

The brain tissue was lysed in RIPA lysis buffer. Protein amounts were determined by BCA protein assay kit. After electrophoresis on 12% sodium dodecyl sulfate-polyacrylamide gel, the total protein was transferred to polyvinylidene fluoride membranes. The membranes were blocked in TBS/0.1% Tween 20 containing 5% skimmed milk powder and incubated overnight at 4°C with the following primary antibodies: NLRP3 (rabbit monoclonal; 1:1,000, Cell Signaling Technology, USA), caspase-1 (rabbit monoclonal; 1:1,000, Abcam, UK), mouse anti-IL-1β (mouse monoclonal; 1:1,000, Cell Signaling Technology, USA), and β-actin (1:5,000). After washing three times, the membranes were incubated with HRP-conjugated IgG secondary antibody (1:1,000) for 2 h at room temperature. The blots were visualized using an enhanced chemiluminescence luminescent substrate (Thermo Fisher Scientific, Waltham, MA, USA) and exposed to X-ray film. The densitometric analysis was performed with a gel image analysis system (Bio-Rad, Hercules, CA, USA) using β-actin as internal control.

### 2.5 ELISA

Brain tissue was ground into homogenate on ice in PBS solution (pH 7.4). The homogenate was centrifuged for 10 min at 5,000×*g* and the supernatant was collected. Levels of TNF-α, IL-1β, and Aβ-42 were measured using mouse ELISA kits (Elabscience, Wuhan, China) according to the manufacturer’s instructions.

### 2.6 Statistical analysis

All analyses were performed using SPSS 19.0 statistical software (IBM, Armonk, NY, USA) and data were presented as mean ± standard deviation (SD). Comparisons between groups were conducted by Student’s *t*-test or one-way ANOVA followed by the least significant difference (LSD) method for multiple comparisons. Two-sided *P* < 0.05 was considered to be statistically significant.

## 3 Results

### 3.1 Acacetin improved spatial learning and memory in AD mice

**Figure 1:**
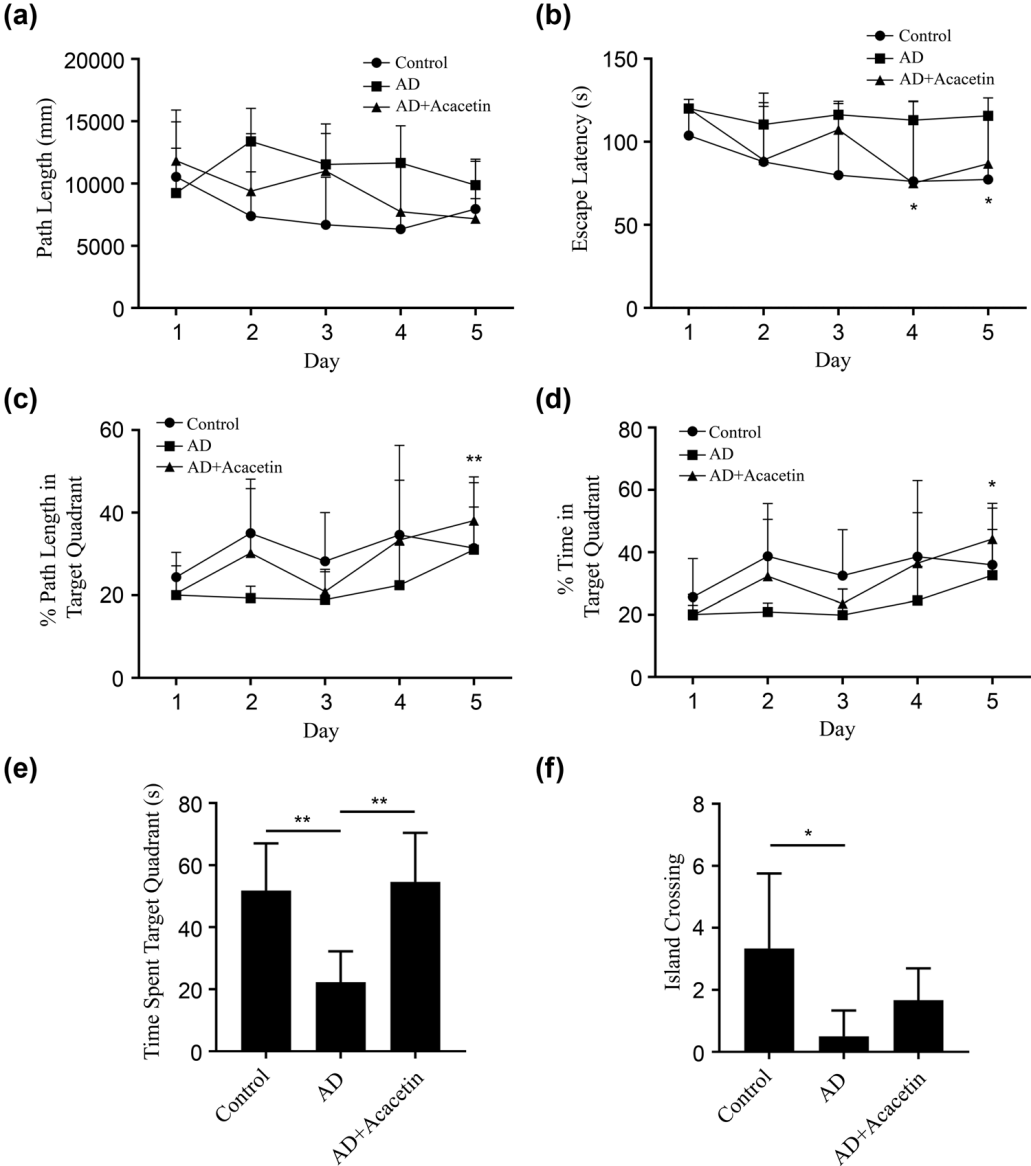
Acacetin ameliorated spatial learning and memory impairment of AD mice in the MWM test. (a–d) Spatial acquisition trials with the hidden platform. (a) Path length to the hidden platform. (b) Escape latency to the hidden platform. (c) Percentage of total path length spent in the target quadrant. (d) Percentage of time spent in the target quadrant. Data are presented as mean ± SD. *N* = 6 mice in each group tested four times a day. Statistical analysis was performed using repeated measures ANOVA with LSD *post hoc* test, *P < 0.05, **P < 0.01 in comparison with Day 1. (e and f) Probe trials with the absence of the platform. (e) Time spent in the target quadrant. (f) The number of platform crossings. Data are presented as mean ± SD. *N* = 6 mice in each group. One-way ANOVA followed by Tukey’s *post hoc* test was used. *P < 0.05 and **P < 0.01.

MWM test was used to detect the effects of acacetin on spatial learning and memory in AD mice. The time and distance of finding the platform under the surface of the water were recorded in the navigation experiment. Compared with the control group and the acacetin group, the AD model group spent more time and longer journey (Figure 1a and b), indicating that the learning ability was decreased. The time of finding the platform in the acacetin group showed a downward trending (compared with Day 1, *P* < 0.05), and the time and the distance of staying in the target quadrant also showed an upward trending. In the probe trial, after removing the platform, the number of times the mice crossed the platform and the time the mice spent in the target quadrant were recorded. The number of times of crossing the platform and the time spent in the target quadrant in the AD model group were significantly less than those in the control group and the acacetin group (Figure 1e and f, *P* < 0.05), indicating that the memory of the AD model group was impaired and the memory persistence was poor, while acacetin could ameliorate the memory impairment of AD mice. The results of MWM showed that acacetin can improve spatial learning and memory in AD mice.

### 3.2 Acacetin reduced SP formation in AD mice

**Figure 2:**
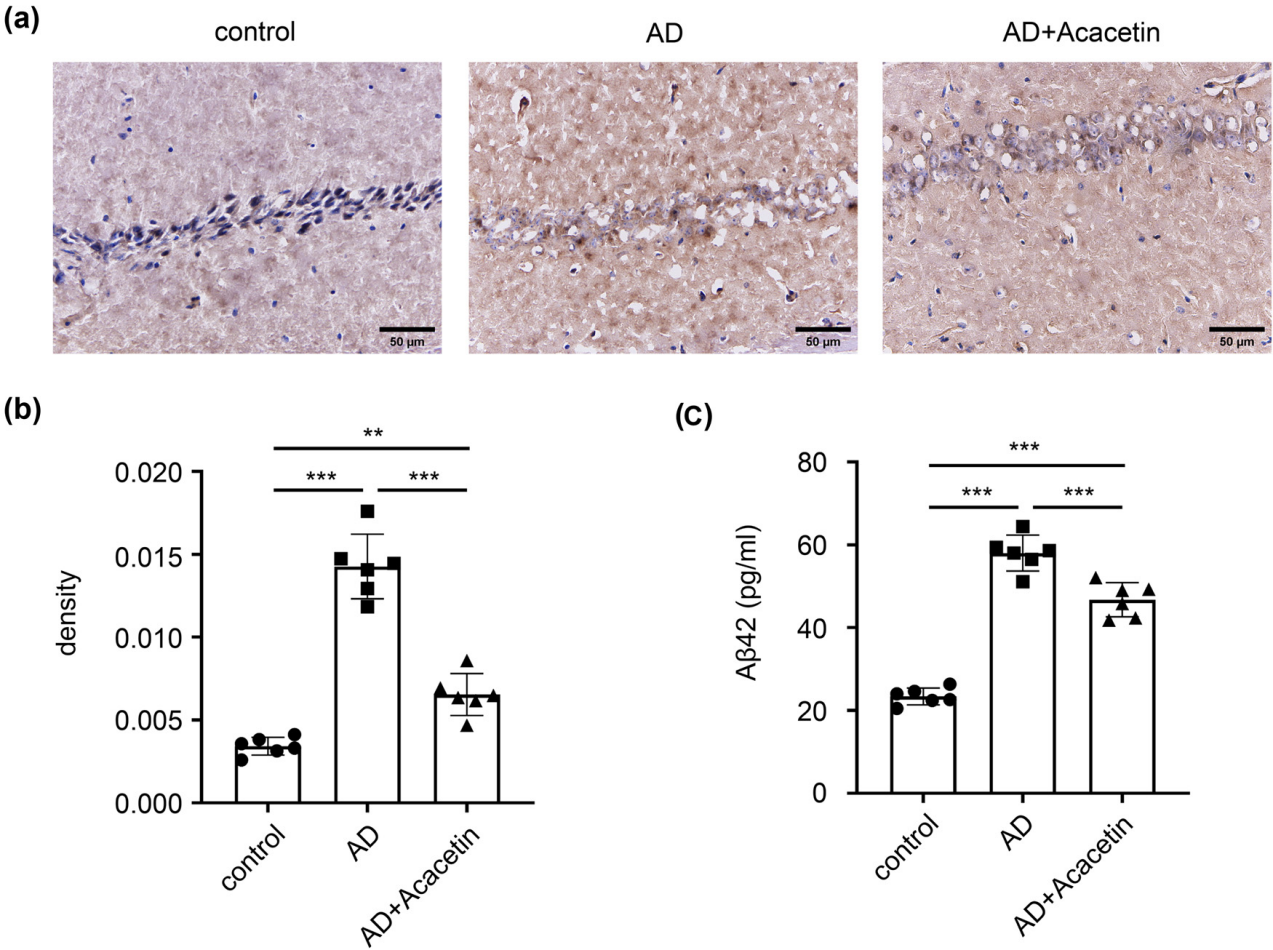
Acacetin decreases the expression of Aβ in AD mice. (a) Immunohistochemistry for APP/β expression in hippocampus of mice brain 400× (scale bar: 50 µm). The nucleus was stained in blue and APP/β was stained in brown. (b) The optical density of APP/β. (c) Levels of Aβ-42 were measured by ELISA. Data are presented as mean ± SD. *N* = 6 mice in each group. One-way ANOVA followed by Tukey’s *post hoc* test was used. *P < 0.05 and **P < 0.01.

The typical pathological feature of AD is SP formed by extracellular Aβ deposition. Results of immunohistochemistry showed that compared with the control group, the number of Aβ deposition in the AD model group was increased. Compared with the AD model group, the number of Aβ depositions was significantly decreased in the acacetin group. Furthermore, results of ELISA showed that Aβ-42 in the AD model group was significantly higher than the control group. Compared with the AD model group, Aβ-42 was significantly decreased in the acacetin group. This indicated that acacetin can reduce the SP formation in AD mice (Figure 2).

### 3.3 Acacetin inhibited NLRP3 signaling pathway in AD mice

**Figure 3:**
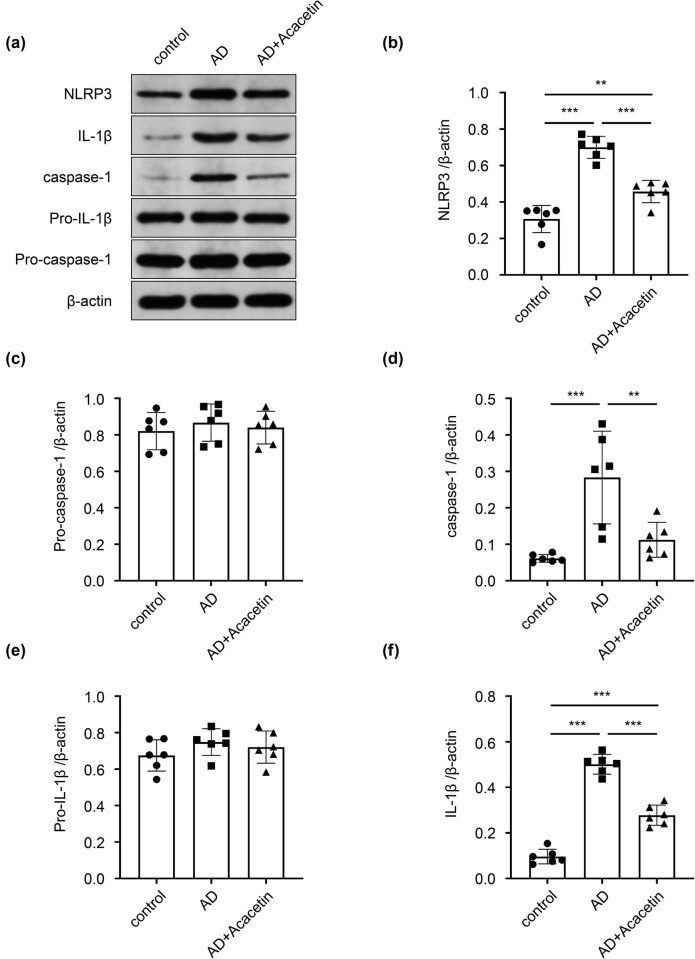
Acacetin prevents NLRP3 signaling pathway in the brain tissue of AD mice. Protein expressions of NLRP3, pro-caspase-1, pro-IL-1β, and IL-1β were measured by WB assay. (a) Representative immunoblots. (b–f) Densitometric analysis of NLRP3, pro-caspase-1, caspase-1, pro-IL-1β, and IL-1β normalized to β-actin. Data are presented as mean ± SD. *N* = 6 mice in each group. One-way ANOVA followed by Tukey’s *post hoc* test was used. *P < 0.05 and **P < 0.01.

Results of WB assay showed that there were no significant differences in pro-caspase-1 and 1 pro-IL-1β among groups. However, compared with the control group, the expressions of NLRP3, caspase-1, and IL-1β in the brain tissue of the AD model group were significantly increased. Furthermore, compared with the model group, the expressions of NLRP3, caspase-1, and IL-1β were significantly decreased in the acacetin group, indicating that acacetin can inhibit the activation of NLRP3 signaling pathway in AD mice (Figure 3).

### 3.4 Acacetin depressed release of inflammatory factors in AD mice

**Figure 4:**
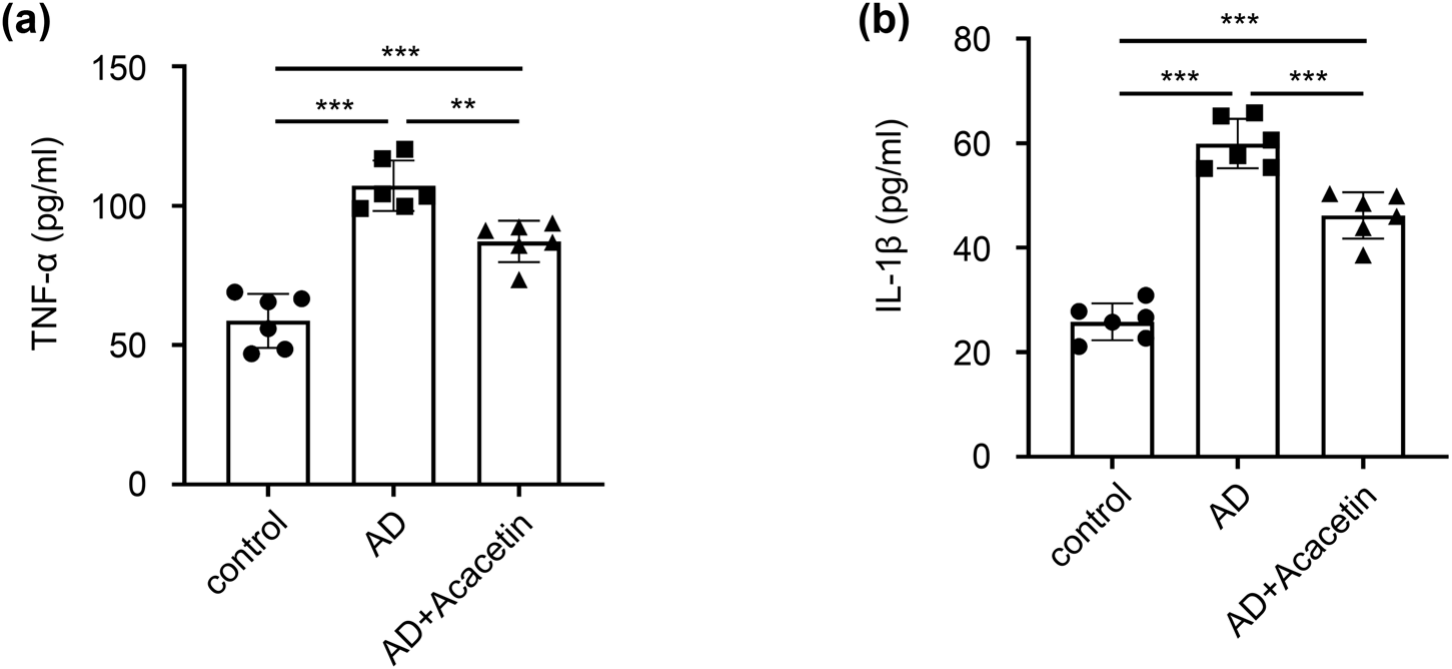
Acacetin inhibits release of TNF-α and IL-1β in the brain tissue of AD mice. Levels of (a) TNF-α and (b) IL-1β were determined by ELISA. Data are presented as mean ± SD. *N* = 6 mice in each group. One-way ANOVA followed by Tukey’s *post hoc* test was used. *P < 0.05 and **P < 0.01.

The results of ELISA showed that there was a little expression of TNF-α and IL-1β in the brain tissue of the control group. Compared with the control group, the levels of TNF-α and IL-1β were significantly increased in the AD model group. However compared with the AD model group, the levels of TNF-α and IL-1β were significantly decreased in the acacetin group. It indicated that acacetin can reduce the release of inflammatory factors in AD mice (Figure 4).

## 4 Discussion

To date, there are few studies that have investigated the association between acacetin and AD. Previous studies have shown that acacetin have a protective effect in Drosophila models of AD. Therefore, we intend to further verify whether acacetin has a protective effect on AD mice [17]. In this study, we used APP/PS1 double transgenic mice (a classic AD animal model) to verify the protective effect of acacetin on AD. Learning and memory impairment is an important clinical manifestation in AD. Thus, we conducted an MWM test to detect the spatial learning and memory in AD mice. The results showed that acacetin can ameliorate the impairment of spatial learning and memory in AD mice.

The production and degradation of Aβ in normal human brain is dynamically balanced. However, excessive Aβ accumulation was detected in the brain of the patients with AD, clearing that Aβ has a protective effect on AD. In our study, the expression of Aβ significantly increased in the brain tissue of AD mice, and acacetin could significantly reduce the Aβ deposition in the brain of AD mice. Thus, it indicated that acacetin could help to clear the excessive accumulation of Aβ and play a protective role in AD.

Several studies have confirmed that NLRP3 inflammasome plays a key role in Aβ accumulation and Tau lesions. Aβ activates NLRP3 inflammasome in microglia through lysosome damage and cathepsin B release, which leads to the release of mature IL-1β [18,19]. Knockout of NLRP3 inflammasome can effectively decrease the deposition of SP [20]. It was also found that the phosphorylation level of Tau and pathological characteristics in NLRP3 deficient mice were significantly lower than those in normal mice, and NLRP3 inflammasome inhibitor MCC950 could reduce the formation of neurofibril tangles induced by Stancu et al. [21]. The aforementioned studies have revealed that NLRP3 inflammasomes are potential targets for the treatment of AD. Our study showed that acacetin can reduce the protein expression of NLRP3, caspase-1, and IL-1β in the brain tissue of AD mice, and downregulate the release of inflammatory factors TNF-α and 1β. It clearly suggested that the protective effect of acacetin on AD is related to the inhibition of NLRP3 inflammasome.

## 5 Conclusion

In summary, acacetin plays a protective role in AD by improving spatial learning and memory and reducing the formation of SP in AD mice. The mechanism may be related to inhibiting the activation of NLRP3 inflammasome and decreasing the expression of related inflammatory factors.
